# Overview of OxLDL and Its Impact on Cardiovascular Health: Focus on Atherosclerosis

**DOI:** 10.3389/fphar.2020.613780

**Published:** 2021-01-11

**Authors:** Anastasia V. Poznyak, Nikita G. Nikiforov, Alexander M. Markin, Dmitry A. Kashirskikh, Veronika A. Myasoedova, Elena V. Gerasimova, Alexander N. Orekhov

**Affiliations:** ^1^ Institute for Atherosclerosis Research, Skolkovo Innovative Center, Moscow, Russia; ^2^ Centre of Collective Usage, Institute of Gene Biology, Russian Academy of Sciences, Moscow, Russia; ^3^ National Medical Research Center of Cardiology, Institute of Experimental Cardiology, Moscow, Russia; ^4^ Laboratory of Cellular and Molecular Pathology of the Cardiovascular System, Institute of Human Morphology, Moscow, Russia; ^5^ Laboratory of Angiopathology, Institute of General Pathology and Pathophysiology, Moscow, Russia; ^6^ Centro Cardiologico Monzino, Istituti di Ricovero e Cura a Carattere Scientifico, Milan, Italy; ^7^ Department of Systemic Rheumatic Diseases, V.A. Nasonova Research Institute of Rheumatology, Moscow, Russia

**Keywords:** oxLDL, atherosclerosis, cardiovascular disease, low-density lipoprotein, oxidative stress

## Abstract

Cardiovascular pathologies maintain the leading position in mortality worldwide. Atherosclerosis is a chronic disease that can result in a variety of serious complications, such as myocardial infarction, stroke, and cardiovascular disease. Inflammation and lipid metabolism alterations play a crucial role in atherogenesis, but the details of relationships and causality of these fundamental processes remain not clear. The oxidation of LDL was considered the main atherogenic modification of LDL within the vascular wall for decades. However, recent investigations provided a growing body of evidence in support of the multiple LDL modification theory. It suggests that LDL particles undergo numerous modifications that change their size, density, and chemical properties within the blood flow and vascular wall. Oxidation is the last stage in this cascade resulting in the atherogenic properties. Moreover, recent investigations have discovered that oxLDL may have both anti-inflammatory and pro-inflammatory properties. Oxidized LDL can trigger inflammation through the activation of macrophages and other cells. After all, oxidized LDL is still a promising object for further investigations that have the potential to clarify the unknown parts of the atherogenic process. In this review, we discuss the role of oxLDL in atherosclerosis development on different levels.

## Oxidative Modification Hypothesis

Cardiovascular disease (CVD) is one of the leading causes of premature death and disability in Europe. According to the World Health Organization (WHO), about 75% of all deaths from CVD can be prevented by following a healthy lifestyle. A healthy lifestyle includes smoking cessation, a healthy diet, and sufficient physical activity (Benjamin et al., 2019).

Brown and Goldstein were the first to postulate that LDL has to undergo some structural changes to achieve atherogenic properties (Brown and Goldstein, 1983). They also figured out that circulating LDL uptake by macrophages is not fast enough to load the cell with cholesterol, which is necessary for the foam cell formation. Moreover, patients totally deficient for the native LDL receptor are still able to store large amounts of cholesterol in their macrophages. Consequently, they suggested that the classic LDL receptor is not involved in the uptake of modified LDL. Goldstein et al. also found the acetyl LDL receptor, i.e., responsible for the modified LDL uptake (Goldstein et al., 1979). Later, this receptor was cloned in Krieger’s laboratory and called scavenger receptor A (Kodama et al., 1988). Several more scavenger receptors were identified. Also, several different LDL modifications that make particles recognizable for scavenger receptors of macrophages are known. Among such modifications, enzymatic modifications are complexing with immunoglobulins and oxidation (Steinberg and Witztum, 1990).

Oxidative stress manifests itself in excessive ROS generation and oxidation of LDL particles. The emergence of oxLDL is crucial for the progression of CVD linked to atherosclerosis. The main factor underlying oxidative stress is a disbalance between radical production (reactive oxygen and/or nitrogen species formation) and radical scavenging systems (the antioxidant defense system) (Leopold and Loscalzo, 2008). Also, hypertension, insulin resistance, diabetes mellitus, hypercholesterolemia, obesity, dyslipidemia, a high level of C-reactive protein (CRP), immunological disorder, vascular wall inflammation, a genetic predisposition, bacterial infection, stress, alcohol consumption, and smoking were all observed to be risk factors for atherosclerosis development (Holvoet, 2012).

OxLDL can trigger the expression of adhesion molecules on the cell surface and thus stimulate the activation of endothelial cells (Obermayer et al., 2018). These adhesion molecules mediate the rolling and adhesion of blood leukocytes, that adhere to the endothelium and then, in response to chemokines, migrate into the intima. As the consequence of the macrophage activation, proinflammatory cytokines are released, ROS are synthesized, and proteolytic enzymes are also produced that contribute to the matrix degradation. This leads to plaque destabilization (Chen and Khismatullin, 2015) Table 1.

**TABLE 1:** The effect of oxLDL on key mechanisms involved in atherogenesis.

Atherogenic process	Involved cells	OxLDL effect	References
Endothelial dysfunction	ECs	OxLDL induces endothelial dysfunction *via* cytoplasmic adapter protein TRAF3IP2	Valente et al. (2014)
NO production	ECs	OxLDL inhibits eNOS activity and NO production	Blair et al. (1999)
Apoptosis	VSMCs	OxLDL overproduction triggers LOX-1 expression and apoptosis	Wu et al. (2017)
Cell adhesion	ECs, leukocytes	OxLDL can stimulate the activation of endothelial cells and the production of adhesion molecules that mediate the adhesion of blood leukocytes, that adhere to the endothelium and migrate into the intima. As the consequence of the macrophage activation, proinflammatory cytokines are released, ROS are synthesized, and proteolytic enzymes that contribute to the matrix degradation are also produced. This leads to plaque destabilization	Chen and Khismatullin (2015); Obermayer et al. (2018)
Proliferation	ECs	OxLDL induces cell proliferation *via* Rho/ROCK/Akt/p27kip1 signaling	Zhang et al. (2017)
Cell death	ECs	OxLDL induces cell death *via* cytoplasmic adapter protein TRAF3IP2	Valente et al. (2014)
NLRP3 inflammasome activation	Macrophages	OxLDL and cholesterol crystal accumulation are important triggers of inflammasome activation. OxLDL can activate NLRP3 inflammasome even without cholesterol crystals. Macrophages uptake oxLDL *via* CD36, which results in the intracellular nucleation of cholesterol crystals in lysosomes	Grebe et al. (2018); Poznyak et al. (2020b)

**FIGURE 1:**
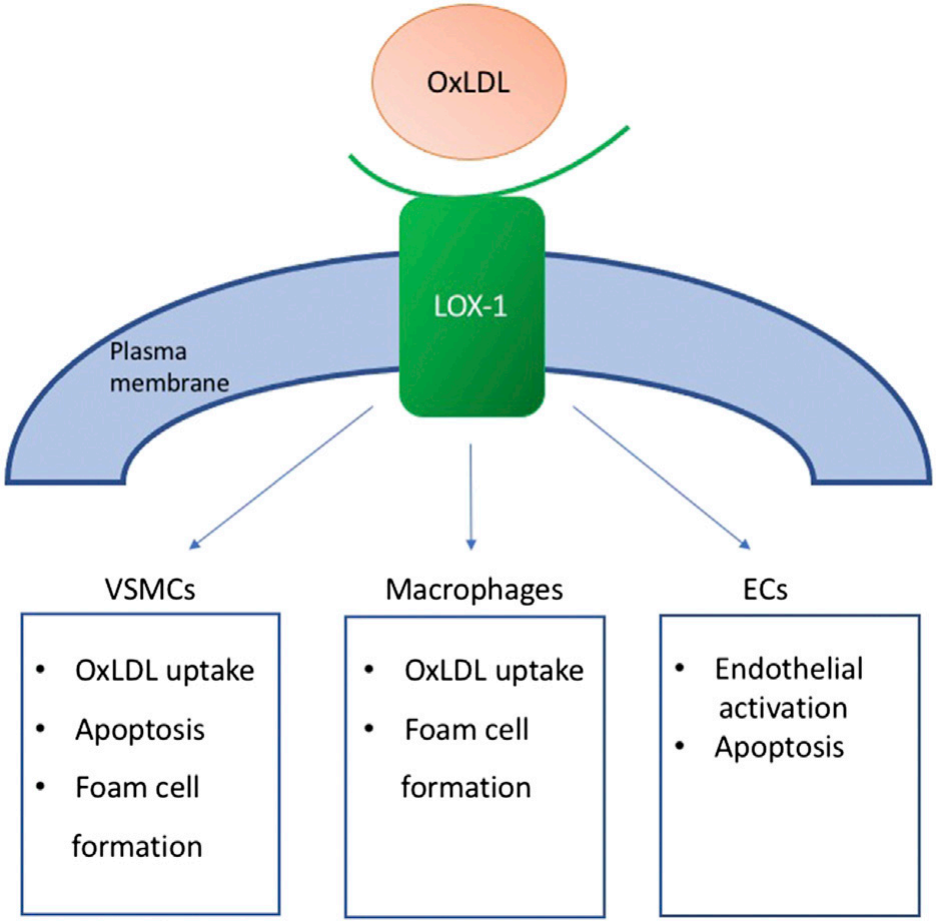
OxLDL binding to LOX-1 triggers various processes in cells of different types. It enhance OxLDL uptake in both macrophages and VSMCs, and stimulate foam cell formation. It also contributes to the endothelial activation and induces apoptosis in VSMCs.

Apart from the scavenger receptors, as SR-A, SR-BI, and CD36, oxLDL also binds to lectin-like oxidized low-density lipoprotein receptor-1 (Zani et al., 2015). LOX-1 consists of a short N-terminal cytoplasmic domain, a transmembrane domain, a neck region, and an extracellular C-type lectin-like extracellular domain. Together they form a type II integral membrane glycoprotein, which was first described as the crucial oxLDL receptor of EC cells (Pirillo et al., 2013). Later, the same receptor was shown on the surface of smooth muscle cells and macrophages. Contribution of LOX-1 to atherosclerosis is briefly summarized in Figure 1.

Under normal conditions, LOX-1 expression is very low, but it can be enhanced in response to proatherogenic or proinflammatory triggers. The LOX-1 level is upregulated in endothelial cells at the early stages of atherogenesis and in advanced plaques. Also, intimal SMCs and macrophages exhibit enhanced expression of LOX-1 in human carotid atherosclerotic plaques (Kattoor et al., 2019). This is potential evidence of the LOX-1 involvement in foam cell formation and activation of the endothelium. Experiments on mice have shown that LOX-1 can also play an important role in the inflammatory response and lipid deposition in heart vessels (Xu et al., 2013).

Overproduction of oxLDL triggers the LOX-1 expression in VSMCs and also triggers apoptosis (Wu et al., 2017). Expression of the proapoptotic protein Bcl-2-associated X protein (Bax) is increased, as well as the expression of the anti-apoptotic protein Bcl2 in response to excessive oxLDL formation (Liu et al., 2005;Toledo-Ibelles and Mas-Oliva, 2018). LOX-1 mediates this effect, and, accordingly, anti-LOX-1 antibodies inhibit the ox-LDL-induced apoptosis. All this data, combined with the colocalization of LOX-1 and Bax in human atherosclerotic plaques, particularly in the rupture-prone shoulder region, provide shreds of evidence of the potential involvement of LOX-1 in the atherosclerotic plaque destabilization (Kataoka et al., 2001).

Lysophosphatidylcholine (LPC) treatment can also increase LOX-1 expression in SMCs. This, subsequently, leads to an increase in oxLDL uptake. Anti-LOX-1 antibodies were shown to inhibit the uptake of oxLDL after the LPC stimulation (Chistiakov et al., 2016). OxLDL affects the NLRP3 inflammasome as well. OxLDL and cholesterol crystal accumulation are important triggers of inflammasome activation, which serves as the link between inflammation and lipid metabolism. OxLDL can activate the NLRP3 inflammasome even without cholesterol crystals. Macrophages uptake oxLDL *via* CD36, which results in the intracellular nucleation of cholesterol crystals in lysosomes. This process activates inflammasome with the same result as when the cholesterol crystals are phagocytosed (Poznyak et al., 2020b).

Oxidative modification has been attracting the attention of researchers for the last two decades. Therefore, it is the most well-investigated LDL modification for now. What is more, antioxidant compounds, including probucol, probucol analogs, vitamin E, coenzyme Q, diphenylphenylenediamine, and butylated hydroxytoluene were investigated in the scope of atherosclerosis and oxLDL in various animal models. Mostly, the results of these investigations were successful (Witztum and Steinberg, 2001). The oxidative modification hypothesis was suggested by Steinberg and Chisolm groups (Hessler et al., 1979; Henriksen et al., 1981; Steinberg and Witztum, 2002). Evidence in support of this theory is provided in Table 2.

**TABLE 2:** Pieces of evidence that support the theory of oxidative modification.

Pieces of evidence that support the theory of oxidative modification	References
LDL oxidation was observed *in vivo*	Palinski et al. (1989)
OxLDL was found in atherosclerotic lesions	Nishi et al. (2002)
Autoantibodies are generated against oxLDL	Wu and Lefvert (1995)
Titers of autoantibodies correlate with the extent of atherosclerosis	Bergmark et al. (1995)
Knocking out scavenger receptors (both scavenger receptor A or CD36) ameliorates atherosclerosis	Moore and Freeman (2006)
Knocking out 12/15-lipoxygenase that can oxidize LDL ameliorates atherosclerosis	Cyrus et al. (1999)

## Oxidative Stress

Oxidative stress is considered an important risk factor for the pathogenesis of various diseases, such as cancer, age-associated disorders, neurodegenerative diseases, autoimmune disorders, and CVD. There is more than one way through which oxidative stress influences the development of CVD. Regarding atherosclerosis, oxidation of LDL particles in the vascular endothelium was reported to be an initial event in the atherosclerotic plaque formation. Thus, phenotypical changes in endothelial cell surfaces are triggered by the production of intracellular ROS. Moreover, superoxide generates cytotoxic peroxynitrite and lowers the activity of nitric oxide. The platelet aggregation is enhanced, and vascular function is modulated by superoxide through vasoconstriction (Laursen et al., 2001; Stocker and Keaney, 2004).

Oxidative stress can be a part of cancer development and the aging process. When the organism ages, the activity of the endogenous defense system lowers, and the ROS generation enhances, leading to the continuous damage of cellular structures. For example, inhibition of paraoxanase activity by high serum levels of anti-beta-2-glycoprotein I (b2GPI) antibodies leads to the enhancement of oxidative stress levels. Paraoxanase has antioxidant features and protects LDL from oxidative injury (Durrington et al., 2001). Endothelial nitric oxide synthases and mitochondrial enzymes are the most active source of endogenous oxidants in aging. Oxidants can able to directly affect nucleic acids and cause mutations, which contribute to the apoptosis suppression and metastasis process stimulation (Halliwell, 2007).

## Enzymes Involved in ROS Generation

ROS formation involves several enzymes and enzymatic systems, such as NADPH oxidase (NOX), uncoupled endothelial nitric oxide synthase (eNOS), lipoxygenases (LOX), and others (Poznyak et al., 2020a).

**FIGURE 2:**
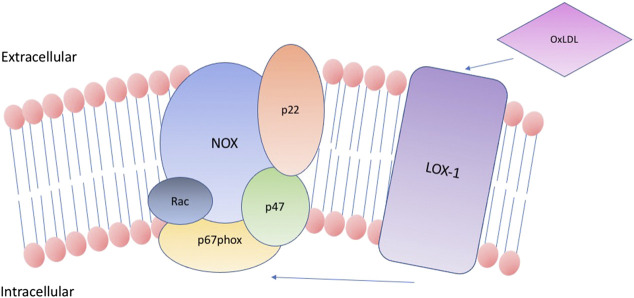
Scheme of NADPH oxidase complex structure. NADPH oxidase consists of two membranes bound subunits, p22 and NOX homologue, and also few cytosolic regulatory subunits (p47, Rac, p67phox).

NADPH oxidases are a group of enzyme complexes that consist of several subunits and generate superoxide from molecular oxygen with the NADPH as the electron donor. P22phox and a Nox homologue, two membranes bound subunits, and also few cytosolic regulatory subunits form an NADPH oxidase complex (Panday et al., 2015). This structure is schematically shown in Figure 2. Nox was first identified to be expressed in the membrane of “professional” phagocytic cells, such as macrophages, neutrophils, monocytes, tissue dendritic cells, and mast cells. These cells produce many ROS to deal with pathogens (Cross and Segal, 2004). Then, NADPH oxidases were also found in other nonphagocytic cell types, among which there are smooth muscle cells (SMCs) and endothelial cells (ECs) (Pendyala et al., 2009). Seven Nox isoforms have been described that express in humans. Among them, only Nox1, Nox2, Nox4, and Nox5 are expressed in the endothelium, vascular SMCs, fibroblasts, or perivascular adipocytes at significant levels. Further investigations prove the role of Nox homologues in the atherogenesis process (Konior et al., 2014).

Despite the belonging of Nox isoforms to the one family, they have a different impact on atherosclerosis development. Thus, Nox1 and Nox2 are considered pro-atherogenic. It is supported by the decreased severity of atherosclerosis that was observed in apoE^−/−^ mice with genetic deletion of Nox1. Nox2 deficiency alleviated atherosclerosis in the descending aorta of model animals. However, this effect was not observed in the aortic sinus (Sheehan et al., 2011). The anti-atherogenic effect was observed in the murine model for Nox4 (Fulton and Barman, 2016).

Interestingly, Nox5 is not presented in the rodent genome that hinders the investigation of the role of Nox5 in atherosclerosis progression. In terms of human atherosclerosis, Nox5 expression level was found to be increased in atherosclerotic lesions (Kigawa et al., 2017).

However, the mechanisms of its action is a matter of doubt.

Hydrogen peroxide and superoxide anions are produced by xanthine oxidases (XO) with the use of molecular oxygen as an electron acceptor. Xanthine oxidases can be found in blood plasma and within the population of endothelial cells. Also, elevated levels of XO were observed in atherosclerotic plaques (Martin et al., 2004). Xanthine oxidase inhibitors were shown to reduce the atherogenesis process in apoE^−/−^ mice. What is more, the endothelial dysfunction in heavy smokers appeared to be decreased by the inhibition of xanthine oxidase (Dopp et al., 2011).

Notably, uric acid is another product of xanthine oxidases, which in high blood concentration may result in clinical manifestation of gout, which, in turn, is associated with the enhanced burden of atherosclerosis-related events. The activity of xanthine oxidases triggers the expression of scavenger receptor CD-36 and lectin-like oxidized low-density lipoprotein receptor-1 (LOX-1) in macrophages and vascular smooth muscle cells, which contributes to the binding of oxLDL particles. Thus, the over-production of uric acid is associated with foam cell formation (Chen et al., 2016).

ENOS produces nitric oxide playing a crucial vasoprotective role for the endothelium, but in pathologies associated with oxidative stress, the functioning of eNOS can be altered (Fleissner and Thum, 2011). Oxidative stress is associated with endothelial dysfunction through the overproduction of superoxide inactivating nitric oxide. Lasting oxidative stress results in eNOS uncoupling (uncoupling of O2 reduction from the NO synthesis), which, in turn, leads to the generation of superoxide instead of nitric oxide. Among the potential causes of uncoupling of eNOS is the deficiency of eNOS substrate (l-arginine), eNOS S-glutathionylation, and the deficiency of eNOS cofactor, tetrahydrobiopterin (BH4). Peroxynitrite, which is a direct product of the reaction between NO and superoxide, can oxidize BH4, and this can result in BH4 deficiency (Förstermann and Li, 2011). In apolipoprotein E deficient mice, increased oxidative degradation of BH4 and eNOS uncoupling could be seen in cardiovascular tissues (Bendall et al., 2014). Evidence of ROS production by uncoupled eNOS has been obtained in patients with atherosclerosis, as well as in subjects with hypercholesterolemia, hypertension, diabetes mellitus, and chronic smokers (Schulz et al., 2011).

## LDL Oxidation

Oxidized LDL is derived from different sources, including metal ions, reactive oxygen species (ROS), lipoxygenase, and myeloperoxidase, and can contain various parts. So, we can propose the following general definition: oxLDL is a particle obtained from circulating LDL that may have peroxides or their degradation products generated within the LDL molecule or elsewhere in the body associated with the particle. At the same time, an exact mechanism of LDL oxidation is still unclear (Levitan et al., 2010; Parthasarathy et al., 2010).

Among types of oxidative modification of LDL particles, there are non-enzyme-mediated modifications, including interaction with proteoglycan, free radical, glycosylation, and modifications mediated by enzymes (oxidase, lipase, myeloperoxidase, and others). Also, depending on the LDL component that underwent oxidation, modifications are divided into lipid component modification and protein component modification (Ivanova et al., 2017). In both cases of enzymatic and non-enzymatic modifications, chemical properties, physical structure, and biological activity of LDL particles can be changed. It was also revealed that macrophages, as well as smooth muscle cells and endothelial cells, can modify LDL (Lusis, 2000).

LDL is a complex particle with variative oxidation sensitivity. Oxidation of LDL is a gradual process, during which oxidation is occurring continuously, from mild to extensive, containing different ratios of various potentially toxic components of oxidized proteins and oxidized lipids. It explains the heterogeneity of the composition, metabolism, and biological characteristics of oxidized LDL particles. Lipid aldehyde and sterol and lipid peroxide are oxidized and exist in different proportions. All types of bioactive lipids of oxidized LDL communicate with molecular targets of the cell through various mechanisms and play physiological or pathological roles, mechanisms of which are still not clearly understood (Parthasarathy et al., 2010).

The history of modified LDL investigations began in 1981 when Henriksen et al. found out that overnight incubation of cultured endothelial cells with native LDL results in the transformation of the latter (Henriksen et al., 1981). LDL obtained specific features that help these particles to be recognized by peritoneal macrophages with high affinity. This transformation is believed to be the main stimuli of the LDL uptake and subsequent foam cell formation (Alique et al., 2015; Linton et al., 2019). Later, it was clarified that LDL undergoes the oxidation process with the co-incubation with cells of various types, such as endothelial cells. This data laid the fundament of the oxidative modification hypothesis of atherogenesis (Itabe et al., 2011). This theory was accepted as the main explanation of the atherosclerotic pathology process. Further investigations provided the results of the animal trials and epidemiological evidence that supported the theory.

The first promising success of the antioxidants uses for atherosclerosis treatment in various animal models resulted in clinical trials that were aimed to prove the oxidative theory at last (Toledo-Ibelles and Mas-Oliva, 2018). Natural antioxidants were among the special interest due to the less amount of adverse effects. Thus, the antioxidant of the first choice was vitamin E. However, these trials appeared not to be so successful (Saremi and Arora, 2010). There are several possible reasons for this. First, the design of the trials did not take into account potential mechanisms of antioxidant effect, potential interaction with other drugs, disease stage, and other important features. Moreover, the difference in the atherosclerosis pathogenesis between various species was not fully investigated.

Oxidative stress is an important trigger of lipid oxidation (Yang et al., 2017). Oxidation is the main modification that makes LDL recognizable for scavenger receptors (SRs) on the surface of macrophages and pericyte-like cells (Zani et al., 2015). After the internalization of large LDL amounts, cells transform into foam cells with the cytoplasm fulfilled with lipids. The emergence of such cells is a specific feature of early atherosclerotic lesions. Nevertheless, native non-modified LDL can trigger the foam cell formation as well, but the concentration has to be about 40 times higher than that of oxLDL (Chistiakov et al., 2017).

Atherosclerosis was believed to develop similarly in humans and model animals, which, at least in part, confused the results of clinical trials. So, antioxidant therapy was shown to be efficient in animals, but not in humans. Despite the disappointing outcome of these trials, several investigators continued to discuss the potential of the oxidative modification hypothesis.

There are several potential reasons for the fail of antioxidant therapy in humans. For example, vitamin E and other used antioxidants can be oxidized within the organism and thus acquire toxic properties instead of beneficial effects, and the properties of their constituents, such as β-, δ-, and γ-tocopherols and tocotrienols for the vitamin E, should be taken into account. Also, the period of the antioxidant use can be too short for the beneficial effect to be observed. Another possible reason is the insufficiency of only one used antioxidant, or, in contrast, an additional antioxidant effect can be unnoticeable on the background of the use of statins, aspirin, and other cardioprotective drugs, which exhibit an antioxidant effect on their own. In all investigations, antioxidants were used only after the disease has manifested itself, while the efficacy of pre-treatment was not assessed. The choice of a suitable antioxidant compound is a big challenge. Thus, the antioxidants that affect mitochondrial oxidation by crossing the membrane of mitochondria may be a better choice than the traditional antioxidants. And, of course, the theory of oxidant origin of atherosclerosis is imperfect and does not consider other mechanisms and pathways involved in atherogenesis.

## Anti-Inflammatory Properties of OxLDL

Interestingly, Lara-Guzman et al. in 2018 demonstrated for the first time that the interaction of THP-1 macrophages and oxLDL could induce eight PGs and eight IsoPs, two of which, PGE1 and 17-trans-PGF3α have anti-inflammatory properties. Moreover, their levels were not enhanced by the treatment of LDL but were significantly induced by oxLDL (Moore and Tabas, 2011). This anti-inflammatory response indicates that the foam cells express pathways to reduce the cytotoxicity and inflammation triggered by cholesterol-loading in macrophages (Lara-Guzmán et al., 2018). Another example of anti-inflammatory consequences of oxLDL action is the interaction of oxLDL with B cells *via* binding to the CD36 receptor. Another example of anti-inflammatory consequences of oxLDL action is the interaction of oxLDL with B cells *via* binding to the CD36 receptor. This leads to anti-oxLDL production, which has anti-inflammatory activity (Rhoads and Major, 2018). This was first described in 1999 by the Witztum group (Hörkkö et al., 1999).

## Multiple Modifications Concept

**FIGURE 3:**
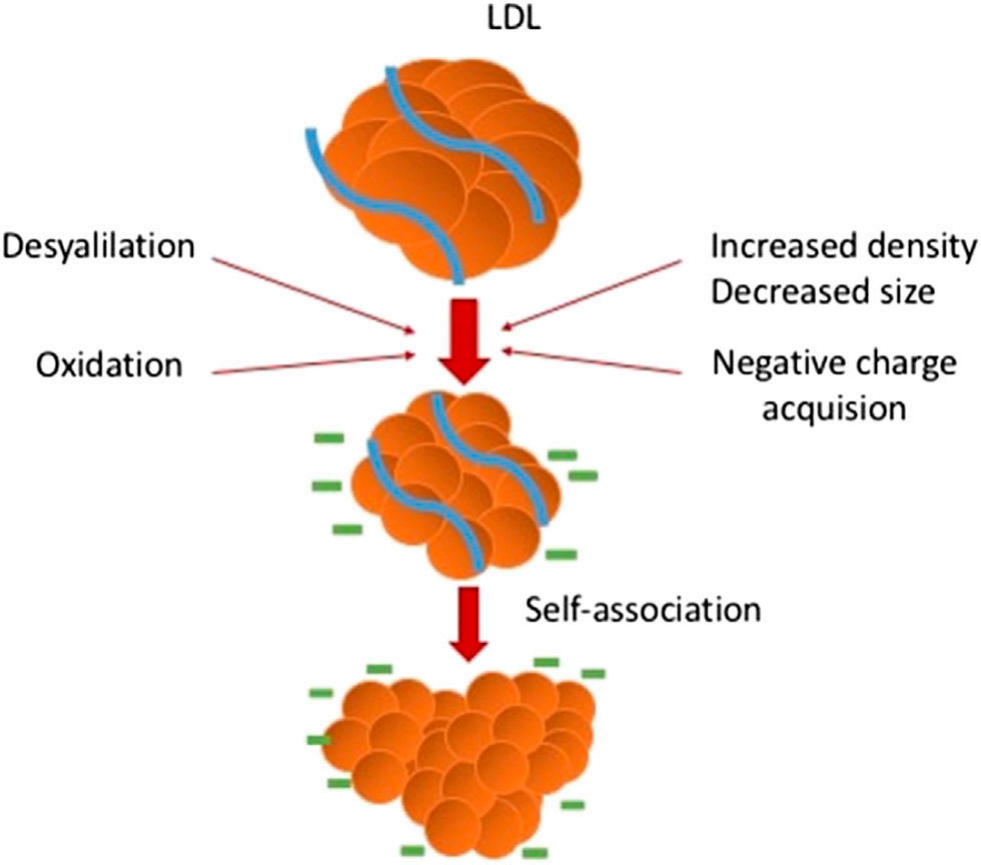
Multiple LDL modifications occurring in the blood and vessel wall.

Through numerous investigations of atherosclerosis, the role of oxidized LDL was postulated as crucial. However, recent findings suggest the conception of multiple LDL modifications occurring within the blood flow (Summerhill et al., 2019). Notably, oxLDL species artificially synthesized *in vitro* have not been detected in the blood. At the same time, signs of oxidations were found among the multiply modified LDL particles. Recent researches show that the negative charge acquiring, size decreasing, glycation, and desialylation can occur with the LDL particles in the blood, as well as other modifications (Figure 3). Particles with all these properties can be found within the circulation of individuals suffering from atherosclerosis and diabetes. However, the modern data suggest that oxidation of LDL can occur not in the blood, but in the arterial wall (Summerhill et al., 2019).

For example, a non-enzymatic reaction of glucose and its metabolites with free amino groups of apoB-100 lysine is known to result in the emergence of glycated LDL (Alipov et al., 2017). Notably, small dense LDL (sdLDL) was demonstrated to be more sensitive to glycation, and glycated LDL is more susceptible to oxidation and formation of glycated LDL and other advanced glycation end products. All these contribute to increased atherogenic properties (Younis et al., 2013). SdLDL is more capable of infiltrating the vascular cells where these particles play a role of cholesterol source. Also, sdLDL is characterized by the decreased affinity for the LDL receptor that contributes to the elongation of the time of circulation. This is one of the reasons for the susceptibility of sdLDL for the other modifications in the blood plasma. Also, sdLDL has an increased affinity to the proteoglycans, which can be found within the intima layer of the arterial wall (Orekhov and Sobenin, 2019; Borén et al., 2020).

Modern understanding of atherosclerosis maintains a particular focus on two processes that have a great significance for disease initiation. These processes are intracellular lipid accumulation, which mostly concerns cholesterol and its esters, and the inflammatory response of the resident arterial cells, which is by the recruitment of circulating inflammatory cells into the subendothelial space of arterial intima where they differentiate into macrophages of the vascular wall. Intracellular lipid accumulation was previously demonstrated to stimulate the cellular processes leading to atherosclerosis development. Multiple modified LDL is generally accepted to be the main source of intracellular cholesterol accumulation (Linton et al., 2019). Atherogenic multiple-modified LDL circulates in the blood of atherosclerosis patients and triggers the intracellular lipid accumulation in the areas that are most prone to atherosclerosis (Orekhov, 2018).

For a long time, the intracellular lipid aggregation was believed to be the trigger of the pro-inflammatory response (Bekkering et al., 2014). Nevertheless, it is clear that intracellular lipid metabolism interacts with the immune response, and both processes are linked to the modified LDL. But the true primary stimuli of this interaction are still uncertain. Truly, cholesterol metabolism variations can impact the inflammatory reactions of macrophages (Moore et al., 2013; Gibson et al., 2018).

Orekhov and colleagues identified ten genes suggested to crucially regulate the formation of foam cells (Orekhov et al., 2020b). Interestingly, none of these genes belongs to the cholesterol metabolism pathways, but seven of them relate to the inflammatory pathway. This finding indicates that the conventional understanding of cholesterol accumulation as the primary event triggering the immune response may not be completely correct. It is more likely that the pro-inflammatory immune response stimulates the cholesterol aggregation within the cell, or at least contributes to this process.

Another important hypothesis is that phagocytosis can play a key role in the interaction between inflammation and cholesterol accumulation. In the classic concept, internalization of the pathogen *via* phagocytosis is an initial event launching the innate immunity reaction. Right after it begins, circulating immune cells are recruited to the focus of possible inflammation by the pro-inflammatory molecules secretion (Tall and Yvan-Charvet, 2015). Modified LDL particles tend to self-associate, and these self-associates have the size quite analogues to the bacterial and other pathogens. Due to this, the phagocytosis of resident subendothelial cells of the arterial wall can be stimulated (Orekhov et al., 2020a).

The following sequence of the initiation of atherosclerosis was proposed:

1. Atherogenic modifications of LDL particles;
2. Modified LDL particles form self-associates;
3. Stimulation of the phagocytosis by the large self-associates;
4. Phagocytosis stimulation leads to the secretion of pro-inflammatory molecules;
5. Secreted pro-inflammatory molecules cause or contribute to the accumulation of intracellular cholesterol (Orekhov et al., 2020b).

This hypothesis helps to clarify the link between the inflammatory response and cholesterol accumulation. Also, it becomes clear that not the accumulation of cholesterol is primary in this sequence of events but inflammatory events. Twelve signaling pathways were analyzed and identified to be regulated in the same way by the interaction of macrophages with the multiple-modified atherogenic naturally occurring LDL and with latex beads, which are the classic stimulant of phagocytosis. That investigation confirmed the hypothesis of the relationships between cholesterol accumulation, phagocytosis, and inflammatory response.

Artificially modified LDL particles were also assessed. Desialylated LDL particles were found to have the most similar to latex beads and naturally circulating LDL in terms of the regulation of signaling pathways. Interestingly, despite the wide use of acetylated and oxidized LDL, LDL with these modifications appeared to be poorly similar to the naturally circulating LDL. Based on this data, oxidized LDL can be concerned as not illustrative enough, at least in the scope of gene regulation investigations.

The role of modified LDL in the stimulation of genetic regulation characteristics in triggering the phagocytosis was established. Also, it was shown that inflammatory molecules having the possibility to affect the intracellular cholesterol accumulation are produced as a result of this regulation. F2RL1, EIF2AK3, and IL15 encoding inflammatory molecules were demonstrated to be linked to the aforementioned signaling pathways. Moreover, these genes are upregulated as a result of the interaction of macrophages with modified LDL (Bergmark et al., 1995). The knockdown of EIF2AK3 and IL15 resulted in the complete inhibition of cholesterol accumulation within macrophages. So it can be concluded that upregulation of at least genes EIF2AK3 and IL15 contribute to cholesterol accumulation. Indeed, EIF2AK3 is directly involved in cholesterol accumulation as it upregulated CD36 and SRA and downregulated on ABCA1, ABCG1, and SRB1 expressions (Guo et al., 2018).

The contribution of IL-15 to the accumulation of lipids needs to be studied further. The inhibition of IL-15 was shown to stimulate atherosclerotic lesion attenuation. Also, IL-15 was reported to be a part of inflammation in adipose tissues leading to obesity-associated metabolic syndrome (Lacraz et al., 2016). PAR2 receptor activation resulted in the inflammatory gene expression enhancement and increased lipid accumulation in macrophages (Hara et al., 2018). Lipid accumulation was not observed to be prevented by the inhibition of PAR2 expression. PAR2 and IL-15 are potentially involved in phagocytosis of aggregated LDL. IL-15 appeared to enhance phagocytosis alone, and together with GM-CSF (Singha et al., 2019), the inhibition of PAR2 by antibodies or knockout leads to inefficient phagocytosis.

## Desialylated LDL Can Be a Better Target Than Oxidized LDL

Apart from the multiple modifications theory of atherosclerosis, it is still important to target the most vulnerable and promising part of the cascade. Various benefits of oxLDL were attracting the attention of researchers for a long time. However, the fail of the oxidative modification hypothesis also led to the revision of the target molecule. Now, desialylation is coming to the stage.

Induction of intracellular cholesterol accumulation was assessed to estimate the atherogenic properties of a lipoprotein particle. A significant reversed correlation between the atherogenic potential of LDL and the sialic acid content of the particles was also observed. The lower was the sialic acid content of LDL the more cholesterol was accumulated by the cells. Other parameters, such as oxidation, oxidation rates, lipid peroxidation products, size, phospholipid content, neutral lipids content, and others, demonstrated no significant correlation with the atherogenicity (Chen et al., 2011; Borén et al., 2020). It makes desialylation the most promising candidate to be the crucial atherogenic modification. It seems that the necessary and sufficient condition for the appearance of atherogenic properties in LDL particles is desialylation.

Moreover, native LDL, in contrast to desialylated LDL, was shown to spontaneously form associates *in vitro* in cell culture conditions. It was shown that the atherogenicity of desialylated LDL correlates directly with the degree of lipoprotein particles’ association. An intracellular cholesterol accumulation can be completely blocked by the removal of the LDL associates formed in culture from the medium. Decreased rate of intracellular degradation and increased phagocytosis uptake of lipoprotein particles results in increased atherogenicity of desialylated LDL self-associates (Chen et al., 2011).

## MicroRNA

MiRNAs represent a group of small non-coding RNAs that bind their target mRNA and thus downregulate gene expression. This regulation mechanism is important for the development of atherosclerosis. Importantly, OxLDL was found to participate in microRNAs regulation (Feinberg and Moore, 2016). Also, oxLDL was shown to suppress important endothelial microRNAs (miRNAs) that modifies endothelial cell homeostasis. These miRNAs played the role of mediators of endothelial injury and inflammation and were also demonstrated to affect macrophages by stimulating lipid accumulation and inflammatory activation (Wang et al., 2017). For example, miRNA let-7g inhibits the expression of the LOX-1 gene, which has an antiatherogenic effect. At the same time, the expression of let-7g is inhibited by oxLDL through the stimulation of transcription factor Oct-1 (Feinberg and Moore, 2016; Wang et al., 2017). In the recent study of Degano et al., 21 miRNA, i.e., up- or down-regulated in response to oxLDL treatment were described (Dégano et al., 2020).

OxLDL can have both pro-inflammatory and anti-inflammatory properties concerning the stage of oxLDL exposure and the degree of oxidation. One of the possible mechanisms underlying the atheroprotective effect of oxLDL may be an oxLDL-triggered miR-29a up-regulation. MiR-29a is important for dendritic cell maturation inhibition (Huang et al., 2016).

OxLDL treatment was shown to upregulate the miR-155, contributing to the decrease in TNF-α, IL-6, and IL-8 secretion (Huang et al., 2010; Li et al., 2016). This is implemented through the reduction of MyD88-dependent NF-κB activation. Another effect of miR-155 is the suppression of scavenger receptors: LOX-1 and SRA in macrophages and LOX-1 and CD36 in dendritic cells. Consequently, lipid uptake amenable with foam cell formation is also suppressed. Moreover, the expression of adhesion molecules (VCAM-1 and ICAM-1) and chemotactic factors (CCL19, CCR21, and CCR7) was also shown to be reduced under the action of miR-155 in dendritic cells and macrophages (Doxaki et al., 2015; Bruen et al., 2019).

Interestingly, a direct angiogenic cytokine, SGC2, which is also crucial for AP-1 regulation, appeared to be a direct miRNA-155 target. Changes in SGC2 expression violate the expression of adhesion molecules (Feinberg and Moore, 2016). Exogenous miR-146a was demonstrated to down-regulate TLR4 and inhibit the activation of TLR4-dependent signaling molecules, among which are FAK, JNK, Pyk2, paxillin, p38 MAPK, ERK1/2, JNK pan, NF-κB, and Src family kinases (Yes, Fyn, Fgr, Lck, Hck, and Lyn) in oxLDL-stimulated macrophages. These effects lead to decreased cholesterol loading and inhibition of inflammatory factors (IL-6, IL-8, MCP-1, CCL2, and MMP-9) expression (Maa et al., 2011).

MiR-146a/b is an important part of the TLR4/MyD88 signaling pathway, in which these miRNAs possibly act as direct inhibitors of IL-1 receptor-associated kinase 1 (IRAK1) and TNF receptor-associated factor 6 (TRAF6) (Saba et al., 2014). IRAK1 and TRAF6 are upstream regulators of IκB kinase (IKK)-mediated NF-κB activation, and also NF-κB can transactivate both genes of miR-146a/b among numerous inflammatory genes, but it is possible to suggest the existence of a negative feedback regulation loop through which miR-146a/b control TLR-mediated inflammation (Pothineni et al., 2017). The results of Chen et al. suggest that miR-146 can, at least in part, regulate the maturation of dendritic cells treated with oxLDL. It seems to be implemented through the selective inhibition of the expression of some surface co-stimulatory molecules, such as CD40, CD80, and CD86, but not CD209 and HLA-DR (Feinberg and Moore, 2016). In dendritic cells, miR-146a was demonstrated to target CD40L (ligand of CD40) mRNA and, consequently, inhibit CD40L-mediated secretion of TNF-α and IL-6. These effects are important for the stimulation of adaptive immune response. Moreover, the interaction between CD40 and its ligand results in the recruitment of TRAF6 and the subsequent activation of NF-κB. This can increase the expression of IL-6, TNF-α, and other pro-inflammatory cytokines, as well as CD40 itself. Thus, miR-146a-mediated inhibition of CD40L is potentially important for the termination of the key positive feedback loop during the maturation of dendritic cells (Chatzigeorgiou et al., 2009).

## OxLDL Antibodies

Numerous proatherogenic features exhibited by oxLDL are potentially caused by oxidized phospholipids comprising oxLDL structure. Oxidized phospholipid products can be recognized by autoantibodies as the principal epitopes (Miller et al., 2011). An idea of the prevention or even treatment for atherosclerosis using antibodies could not have gone unnoticed by investigators. Two main strategies are considered. The first one implies an active immunization with oxidized or native LDL or derivatives. The second comprises the passive administration of anti-oxLDL antibodies (Samson et al., 2012). Atheroprotective properties were shown for both of these strategies in mice and rabbit models. Notably, the efficacy of an active immunization can be mainly the result of augmented cellular immunity (Wolf and Ley, 2019).

Interestingly, anti-pneumococci antibodies exhibited reactivity with pneumococci as well as with minimally oxidatively modified LDL (Shekhar et al., 2018). Moreover, pneumococcal immunization resulted in decreased atherosclerosis development rate in the low-density lipoprotein receptor (LDLR)-deficient mice (Shekhar et al., 2018). The results of the 5-years follow-up from the large trials of the pneumococcal polysaccharide vaccine efficacy for the primary prevention of acute coronary syndromes and ischemic strokes are on the way. This investigation called AUSPICE (Australian Study for the Prevention through Immunization of Cardiovascular Events) involved 55–60 years old human subjects (Ren et al., 2016). A human phase I-IIa of GLACIER (Goal of oxidized LDL and ACtivated macrophage Inhibition by Exposure to a Recombinant antibody) is another trial involved subjects suffering from stable carotid artery or aortic disease. Subjects received placebo or recombinant antibody, MLDL1278A. Inflammatory activity was analyzed with FDG-PET three months after the treatment. However, the results of these trials were unsuccessful (Nilsson and Hansson, 2020).

There are no available data on particular side effects that can accompany the result of immunization against atherosclerosis every time. The most alarming is the uncontrolled immune reactions and alterations of the normal lipid metabolism. It is beyond doubt that the possibility of such side effects should be considered in clinical trials.

## Conclusion

Oxidative stress is an important part of atherosclerosis pathogenesis. It involves the improper balance between ROS production and the activity of antioxidant systems. Thus, numerous enzymatic systems participate in oxidative stress development and resolution. Oxidizing of LDL particles is one of the consequences of alterations in the functioning of both prooxidant and antioxidant systems. Moreover, oxidation of LDL particles in the vascular endothelium was reported to be an initial event in the atherosclerotic plaque formation.

LDL oxidation is undoubtedly an important atherogenic modification of native LDL, that takes place mostly in the vascular wall. Oxidation has been believed to be the only modification, i.e., responsible for the atherogenic properties of LDL particles for decades. However, modern findings provide a growing body of evidence suggesting the existence and importance of numerous LDL modifications that affect both the physical and chemical properties of particles. All these modifications form a cascade, all steps of which contribute to atherosclerosis development.

Even though oxidation is not the only atherogenic modification of LDL, it still has numerous different functions in the development of atherosclerosis. What is more, it has recently been shown that oxLDL may exhibit both anti-inflammatory and pro-inflammatory properties according to the stage of oxLDL exposure and the degree of oxidation.

OxLDL was demonstrated to be involved in the foam cell formation and stimulation of the immune response. OxLDL acts through various mechanisms, among which are interaction with scavenger receptors, modulating inflammation-related molecular pathways, including miRNA regulation and NLRP3 inflammasome activation.

Despite the significant role of oxidized LDL in atherogenesis, the understanding of the disease initiation and development progresses, and a new theory was formulated. It was shown that there are multiple modifications of LDL that allow particles to get the atherogenic properties, and the conception of multiple modifications was suggested. Moreover, traditional attitudes have been changed completely due to the recent findings concerning the event sequence in the atherosclerosis initiation.

Taken together, all available data on oxidized LDL indicates the importance of oxLDL as a therapeutic target. The most promising is the use of anti-oxLDL antibodies by different strategies, including active immunization with oxidized or native LDL or derivatives and passive administration of anti-oxLDL antibodies.

## Author Contributions

Writing—original draft preparation, AP; writing—review and editing, AM, DK, VM, EG, NN, AO.

## Funding

This research was funded by the Russian Science Foundation, grant number 20-15-00337.

## Conflict of Interest

The authors declare that the research was conducted in the absence of any commercial or financial relationships that could be construed as a potential conflict of interest.
